# From DYMUS to DYPARK: Validation of a Screening Questionnaire for Dysphagia in Parkinson’s Disease

**DOI:** 10.1007/s00455-021-10332-1

**Published:** 2021-07-15

**Authors:** Carlotta Dagna, Micol Avenali, Roberto De Icco, Marialuisa Gandolfi, Claudio Solaro, Domenico Restivo, Michelangelo Bartolo, Francesca Meneghello, Giorgio Sandrini, Cristina Tassorelli, DYPARK SIRN Group, M. Berlangieri, S. Cristina, E. Alfonsi, E. Monti, M. Tinazzi, G. Busselli, C. De Paoli, N. Smania, K. Inglese, S. De Santi, N. Taiocchi, B. Zucchelli, G. Ronzoni, S. Nordio, P. Tonin

**Affiliations:** 1 Neurorehabilitation Unit, IRCCS Mondino Foundation, Pavia, Italy; 2 Department of Brain and Behavioral Sciences, University of Pavia, Via Mondino 2, 27100 Pavia, Italy; 3 Department of Neurosciences, Biomedicine and Movement, Neuromotor and Cognitive Rehabilitation Research Center (CRRNC), University of Verona, Verona, Italy; 4 Department of Rehabilitation, C.R.R.F. “Mons. L. Novarese”, Moncrivello, Italy; 5 Department of Neurology, Garibaldi Hospital, Catania, Italy; 6 Neurorehabilitation, Department of Rehabilitation and Advanced Technologies, Habilita Hospital, Zingonia di Ciserano, Italy; 7 IRCCS Ospedale San Camillo, Venezia, Italy

**Keywords:** Dysphagia, Parkinson’s disease, Deglutition disorders, Screening

## Abstract

Dysphagia is a common debilitating symptom in people with Parkinson’s Disease (PD), adequate screening of swallowing disorders is fundamental. The DYMUS questionnaire has shown very good characteristics for the screening of dysphagia in Multiple Sclerosis, and it might also prove useful for screening dysphagia in PD. The primary aim was to test and validate the DYMUS questionnaire in PD patients. This is an observational multicentric study involving 103 patients affected by PD. All subjects filled in the DYMUS and the Eating Assessment Tool (EAT-10) questionnaires. A subgroup of patients (*n* = 53) underwent a fiber-optic endoscopic evaluation of swallowing (FEES) and their dysphagia was scored by means of the Dysphagia Outcome Severity Scale (DOSS). DYMUS showed a relatively high level of internal consistency (Cronbach’s alpha 0.79). A significant positive correlation was found between the DYMUS and the EAT-10 scores (*p* < 0.001), while a negative correlation was found between the DYMUS and the DOSS scores (*p* < 0.001). DYMUS showed a good sensitivity and specificity compared to FEES for detecting dysphagia (area under the curve: 0.82, *p* < 0.001). The ROC curve analysis showed that a DYMUS score ≥ 6 represents a reliable cut-off for the risk of dysphagia. The DYMUS questionnaire proved to be a reliable screening tool to detect dysphagia in patients suffering from PD. It is easy to understand, it can be self-administered and therefore adequate for adoption in the clinical practice with the more convenient name of DYPARK.

**Supplementary Information:**

The online version contains supplementary material available at 10.1007/s00455-021-10332-1.

## Introduction

Parkinson’s Disease (PD) is a progressive neurodegenerative disorder primarily characterized by the hallmarks of motor symptoms, such as tremor, bradykinesia, rigidity, and postural instability. Beside these cardinal symptoms, dysphagia is frequent and clinically relevant [1]. The vast majority of PD patients develop swallowing impairment during the course of their disease and more than 50% of those who are subjectively asymptomatic for dysphagia show swallowing dysfunction when assessed with fiber-optic endoscopic evaluation of swallowing (FEES) or videofluoroscopic swallowing study (VFSS) [2, 3]. It is estimated that up to 80% of patients with early-stage PD experience oropharyngeal dysphagia. At advanced stages, the incidence may be as high as 95% [2, 4].

The presence of swallowing dysfunction in PD patients can be very disabling and have a relevant impact on the quality of life [5]. Dysphagia also leads to insufficient medication intake, malnutrition and aspiration with subsequent pneumonia, which is a major cause of death in PD patients [6]. Although a frequent and debilitating symptom, dysphagia is still underestimated and overlooked in clinical practice. This is possibly due to multiple causes. First of all, swallowing disorders are subjectively reported by only one third of parkinsonian patients [7], a mismatch that likely reflects the fact that patients tend to underreport swallowing difficulties unless specifically questioned about it. Secondly, the careful clinical evaluation of dysphagia requires resources (both in terms of time and of trained professionals) and methodologies (FEES, VFSS, etc.) that are not widely available.

In this frame, screening assessment focused on identifying risk factors are highly needed, in order to identify early patients who should be referred to further, more specific, clinical and/or instrumental investigations. In 2009 the task force of the Movement Disorders Society (MDS) assessed clinical rating scales for PD by means of systematic reviews [8]. Two dysphagia scales, the Swallowing Disturbance Questionnaire (SDQ) and the Dysphagia-Specific Quality of Life (SWAL-QOL) scale, met the criteria for being “suggested” but not the “recommended”. The lower strength of endorsement was motivated by methodological limitations: SDQ was tested on a small size of PD patients, while the SWAL-QOL was tested for dysphagia in neurodegenerative diseases in general, but not specifically in PD. Among available screening questionnaires, the DYMUS has already been validated for the early screening of dysphagia in Multiple Sclerosis [9, 10]. DYMUS is composed by 10 items that require a binary response and has proved to be easily self-administered. It is useful to preliminary select patients who should be referred for more specific and comprehensive clinical and instrumental assessments and, if required, to rehabilitation [9].

The primary aim of this study was to validate the DYMUS questionnaire in PD patients. The secondary aim was to compare the performance of DYMUS in screening dysphagia with the Eating Assessment Tool (EAT-10) [11] and with the instrumental assessment by FEES.

Our hypothesis is that DYMUS may be a reliable and easy-to-use tool to detect dysphagia in PD patients.

## Materials and Methods

This is a prospective multicentric, cross-sectional, observational study conducted in 5 Italian Neurological/Neurorehabilitation Centers (Pavia, Verona, Catania, Zingonia and Moncrivello) between January 2017 and June 2019. The protocol was approved by the local Ethics Committees and patients signed the informed consent before being enrolled in the study.

Patients with PD were included in the study if they satisfied the MDS Clinical Diagnostic Criteria for PD [12] and they had an age range between 40 and 85 years.

Exclusion criteria were:

- Concomitance of other neurological diseases or any clinical conditions potentially causing dysphagia;
- Presence of percutaneous endoscopic gastrostomy (PEG);
- Psychiatric disorders;
- Moderate-severe cognitive impairment (MMSE ≤ 21).

The patients were recruited unselectively and consecutively in each center and underwent examination in a single visit, irrespective of self-reported or doctor-suspected swallowing symptoms.

The patients underwent anamnestic evaluation: diagnosis, disease duration, pharmacological treatment, height and weight were noted.

The clinical stage of disease was defined according to the modified Hoehn and Yahr staging system, global motor disability was evaluated by the MDS-Unified Parkinson’s Disease Rating Scale.

### The Questionnaire

All the patients were asked to fill in the DYMUS and the EAT-10 questionnaires. We also asked the caregiver of each patient to fill in the DYMUS separately.

DYMUS is composed by 10 items, all the answers are dichotomous, coded as 1 or 0, depending on the presence or absence of the event (range of score: 0–10; higher = worse) [1] (Supplementary figure).

EAT-10 is a clinical scale used to document dysphagia symptoms severity in patients with swallowing disorders [11]. EAT-10 includes 10 questions, each scored from 0 to 4 (“no problem” to “severe problem”); the total score is calculated by adding the scores of each question, with a higher score indicating higher self-perceived dysphagia severity.

### Instrumental Examination

For the comparison of DYMUS with FEES, patients enrolled in the centers where FEES was available underwent the instrumental evaluation after completing DYMUS and EAT-10. Based on FEES findings, dysphagia severity was rated according to the Dysphagia Outcome Severity Scale (DOSS) [13]. FEES was performed by an experienced otolaryngology specialist. The images of the structures assigned to the swallowing function were evaluated directly through the lens of the fiber-optic laryngoscope head. During the execution of the exam, the patient was in the upright seated position. Only in a few cases, was it necessary to perform the examination with the patient in a semi-seated position. The procedure generally did not require any preparation. The instrument was inserted transnasally through the path of least resistance, generally along the nasal floor or between the inferior and middle turbinate. For the study of the swallowing act, the distal end of the fiberscope was positioned above the epiglottis at the level of the uvula. This “high” position allows a good visualization of the base of the tongue, of the lateral and posterior pharyngeal walls, of the epiglottis, of the vallecula, of the larynx and of the piriform sinuses. The observation point is ideal for assessing airway closure during swallowing. The swallowing test was carried out with foods of different consistencies, from liquid to solid. More specifically, we recorded 3 swallows with a 2-ml volume of liquid, 3 swallows with 1-ml volume of semisolid and 3 swallows of solid bolus (one bite of biscuit-type food).

Based on endoscopic results, patients were classified using the DOSS into seven levels, subdivided into three different degrees of swallowing function:

- grade 0 = no dysphagia, no diet limitations (levels 6 and 7);
- grade 1 = diet with limitations (levels 3, 4 and 5);
- grade 2 = nutrition with the enteral route (levels 1 and 2).

A score ≤ 5 at the DOSS was used to separate dysphagic from non-dysphagic patients.

FEES is a minimally invasive test that represents the non-radiologic gold standard to verify the severity of dysphagia and is necessary to determine any limitations in the diet.

An additional group of 20 PD patients who were hospitalized in the Neurorehabilitation Unit of the Pavia Centre were evaluated to assess the test–retest validity of the DYMUS scale 1 week apart. None of these patients underwent speech or swallowing therapy during this period, nor were they allowed to change their pharmacological treatment.

## Statistical Analysis

Statistical analysis was performed using “R Statistics Software”. Demographic and clinical characteristics were expressed as mean ± standard deviation. The distribution of DYMUS, EAT-10 and DOSS scores was not normal, and data are therefore presented as median and interquartile range.

For the analysis of the internal and construct validity and reliability of DYMUS we calculated:

- the Cronbach’s alpha coefficient (of the entire questionnaire and subsequently by removing one item at a time). The value of 0.7 was considered the cut-off for defining a relatively high internal consistency;
- the Cohen Kappa (of the entire questionnaire and of each item) calculated for the test–retest of 20 patients.

Correlation coefficient and logistic regression were used to compare the DYMUS score with EAT-10 or DOSS scores. The sensitivity and specificity for the association between DYMUS and EAT-10 and DYMUS and DOSS scales were calculated.

We also computed the correlation coefficient to analyze the DYMUS scores obtained when the questionnaire was filled in by the patients or by their caregivers.

We considered statistically significant a *p* value < 0.05.

Finally, we sought the cut-off score for a reliable identification of potentially dysphagic patients who are candidate for instrumental investigations.

## Results

We enrolled a total of 103 PD patients in the study: 56 males (54.4%) and 47 females (45.6%). The mean age was 68.0 ± 2.8 years (range 40–83 years), the mean MMSE score was 24.9 ± 8.5 and the mean disease duration was 11.5 ± 4.9 years (range 1–31 years) (Table 1). Fifty-one patients underwent FEES evaluation. The median score at the DYMUS Questionnaire was 2 (IQR 1;4), at EAT-10 2 (IQR 0;5) and at DOSS 6 (IQR 5;7).

**Table 1:** Demographic and clinical characteristics of the population enrolled in the study

Variable	Value
N. Subjects Sex (M/F)	103 56/47
Age (years), mean ± SD	68.0 ± 2.8
Disease duration (years), mean ± SD	11.5 ± 4.9

To assess the validity of DYMUS, we evaluated preliminarily the reliability of the Questionnaire using the Cronbach’s alpha coefficient. The 10-item DYMUS questionnaire showed a relatively high internal consistency with a Cronbach’s alpha of 0.79. The coherence analysis was also repeated by calculating Cronbach’s alpha while eliminating an item at a time: the scores obtained confirmed the internal consistency (Table 2).

**Table 2:** Cronbach’s alpha for each item of DYMUS: Coherence analysis repeated by calculating Cronbach’s alpha when eliminating one of the 10 items at a time

Item	Cronbach’s alpha
1	0.7560596
2	0.7847757
3	0.7668145
4	0.7899099
5	0.7726620
6	0.7516790
7	0.7608186
8	0.7738234
9	0.7639014
10	0.7961238

As regards test–retest reliability, the Cohen Kappa of the entire questionnaire was 0.69, which indicates a substantial agreement between the two administrations. We also calculated the Cohen Kappa for each item and found substantial repeatability in 8 out of the 10 items, moderate for items 6 and 7 (Table 3).

**Table 3:** Cohen Kappa for test–retest reliability of the DYMUS items

Item	Cohen kappa
1	0.69
2	0.57
3	0.69
4	0.77
5	1
6	0.48
7	0.47
8	0.68
9	0.69
10	0.78

As regards the score obtained when the DYMUS questionnaire was filled in separately by the patients and their caregivers, we did not find statistically significant differences as regards the total score and the individual items. When analyzing the relation between the scores obtained with the two groups (patients and caregivers), we found a significant positive correlation (*p* value < 0.001 *R* 0.68).

### Correlations Between DYMUS and EAT-10 Scores, and DYMUS and DOSS Scores

**Fig. 1:**
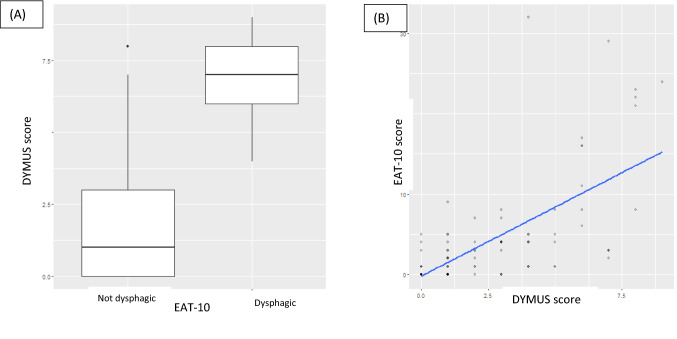
Correlations between EAT-10 and DYMUS scores: we found a significant positive correlation between the DYMUS and the EAT-10 scores (*p* < 0.001 *R* 0.66). **A** Box-plot; **B** scatter-plot

**Fig. 2:**
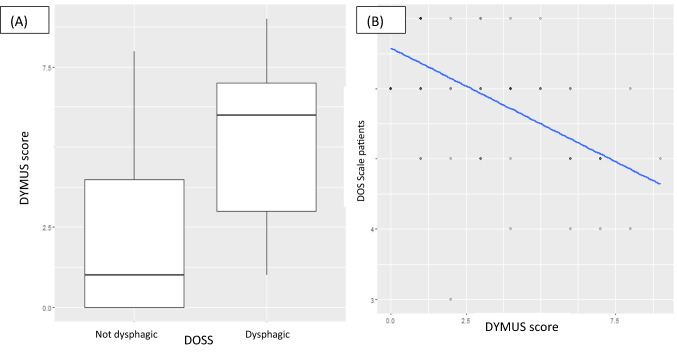
Correlations between EAT-10 and DOSS scores: we found a significant negative correlation between the DYMUS and the DOSS scores (*p* < 0.001 *R* − 0.58). **A** Box-plot; **B** scatter-plot

We found a significant positive correlation between the DYMUS and the EAT-10 scores (*p* < 0.001 *R* 0.66) and a negative correlation between the DYMUS and DOSS scores (*p* < 0.001 *R* − 0.58) (Figs. 1, 2).

The DYMUS proved capable of detecting dysphagic patients (area under the curve: 0.95, *p* < 0.001).

**Fig. 3:**
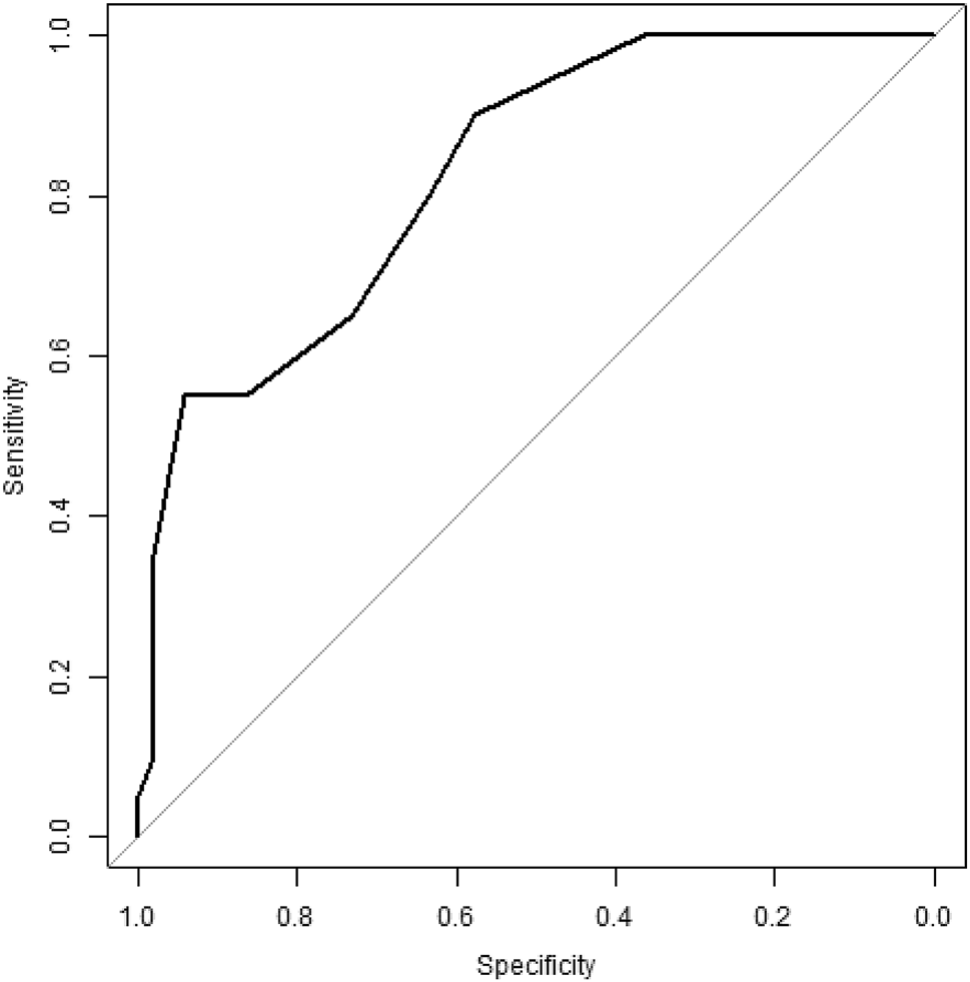
ROC curves for the ability of the DYMUS Scale to identify PD patients with an increased risk of dysphagia. We identified the DYMUS score of 6 as the cut-off for identifying patients at higher risk of dysphagia with a sensibility of 0.7 and a specificity of 0.84

When the DYMUS questionnaire was anchored to DOSS score, we found a DYMUS score of 6 as the cut-off for identifying patients at higher risk of dysphagia with a sensibility of 0.7 and a specificity of 0.84 (Fig. 3).

## Discussion

The main finding of this study is that the DYMUS questionnaire proved a useful screening tool to detect dysphagia in PD patients. Furthermore, it showed a reliable performance when compared to other clinical (EAT-10) and instrumental (FEES) dysphagia assessments.

The presence of swallowing dysfunction in PD patients can be very disabling, negatively affect their quality of life and, in some cases, lead to a fatal outcome [1, 4–6]. Dysphagia may manifest with several symptoms, e.g., difficulties in bolus formation, residual food in oral cavity, multiple swallow, and/or signs, e.g., increased oral transit, decreased swallowing reflex, reduced pharyngeal and oesophageal motility, oesophageal sphincter dysfunction [4–6, 14], which unfortunately are frequently underreported by patients or may go undetected by physicians.

In this context, the early detection of dysphagia in PD patients is of the utmost importance to reduce the risk of complications such as malnutrition and aspiration pneumonia. The diagnostic process and management of dysphagia in PD may require, especially in advances cases, a complex instrumental approach that encompasses VFSS, FEES, electrophysiological investigations and, possibly, the study of the oesophageal motility [15–17]. These investigations are minimally invasive, but often not readily accessible and require expert operators in order to ensure reliability, which makes them non-suitable for the wide-scale use in the daily clinical practice. Hence the importance of a reliable tool for screening patients at risk of dysphagia. The use of such an instrument would prompt the identification of the subjects who actually deserve being referred for more specific investigations.

In the past years, several tests have been proposed for screening dysphagia in PD patients, but these presented some limits. Some of the screening tests have been validated for neurogenic dysphagia in general, not specifically for PD, such as the Sydney Swallowing Questionnaire [18]. Other proposed tests required clinical or instrumental evaluation, such as respectively the Functional Dysphagia Scale [19] and the Swallowing Clinical Assessment Score in Parkinson’s Disease (SCAS-PD) [20].

Finally, very recently two different tools for dysphagia screening in PD failed to show a reliable performance as compared to a non invasive swallowing-respiration assessment system or to FEES, respectively [21, 22]. Among the screening tests, the DYMUS had been already validated for another chronic and progressive neurological disorder, multiple sclerosis [9, 10].

In the present study, we demonstrated that DYMUS has a relatively high internal consistency when used in PD patients (Cronbach’s alpha: 0.79). It also has a substantial repeatability. Only in the DYMUS items that tests swallowing of solid food, we obtained a moderate level of repeatability. A possible interpretation of this finding is that swallowing of solid boluses may be more affected by motor fluctuations than swallowing of liquids. We took care to administer DYMUS in the ON phase during both test and retest sessions, but it is very difficult to fully control for motor performances in sessions taken 7 days apart. Then we compared DYMUS with EAT-10, a validated test for dysphagia screening in neurological patients. For its dichotomic type of responses, we hypothesized that DYMUS could more easily understood by subjects as compared to EAT-10, which has more degrees of variability and requires a subjective evaluation of severity. We also compared DYMUS with DOSS, whose score is based on FEES evaluation.

In doing so, we were able to identify a cut-off score of DYMUS for discriminating PD subjects who are at higher risk of dysphagia. When this cut-off was adopted, DYMUS Questionnaire, compared with the DOSS, showed a good specificity (84%) and sensitivity (70%) to identify patients at higher risk of dysphagia. Hence, DYMUS allows to screen subjects for whom further evaluations is highly recommended.

Another interesting aspect that emerged from our study is the significant correlation of scores when DYMUS was filled in by patients or by their caregivers: this observation has important implications in the clinical practice because it suggests the possibility to use the caregiver as a proxy rater in those conditions where the patient’s reliability may be compromised, i.e., subjects with cognitive impairment.

In this study we focused on PD and therefore, at present, we do not know whether DYMUS is also suitable for atypical parkinsonisms, where dysphagia may be present at disease onset or in the very early phases [22, 23].

In conclusion, DYMUS proved a reliable tool to detect dysphagic PD subjects. It is also easy to self-administer or can be filled in by the caregiver. These characteristics make DYMUS a good tool for the adoption in the daily clinical practice for dysphagia screening, possibly with the more captivating acronym of DYPARK (DYsphagia in PARKinson’s disease).

## Supplementary Information

Below is the link to the electronic supplementary material.

Supplementary file1 (JPG 60 kb)
