# Supplementary figures and images for: From DYMUS to DYPARK: Validation of a Screening Questionnaire for Dysphagia in Parkinson’s Disease
Source: Dysphagia. 2021 Jul 15;37(4):824–30. doi: 10.1007/s00455-021-10332-1 (PMC9345821; doi:10.1007/s00455-021-10332-1)

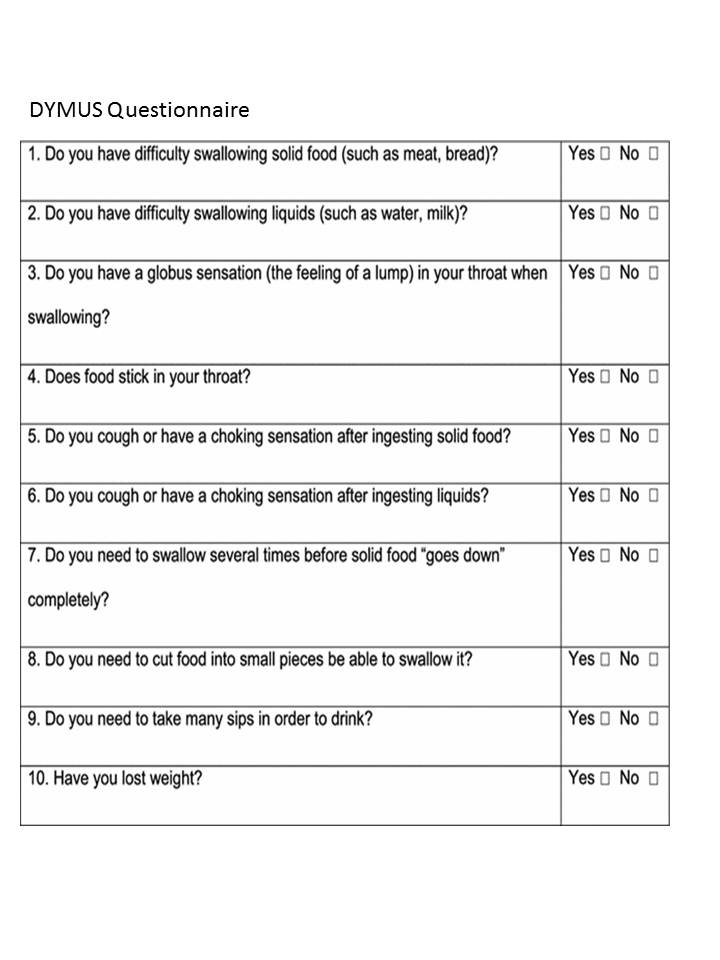

Supplement: Supplementary file 1 — Supplementary file1 (JPG 60 kb) [file 455_2021_10332_MOESM1_ESM.jpg]
